# Screening for opioid use disorder and co-occurring depression and post-traumatic stress disorder in primary care in New Mexico

**DOI:** 10.1186/s13722-023-00362-5

**Published:** 2023-01-27

**Authors:** Cristina Murray-Krezan, Alex Dopp, Lina Tarhuni, Mary D. Carmody, Kirsten Becker, Jessica Anderson, Miriam Komaromy, Lisa S. Meredith, Katherine E. Watkins, Katherine Wagner, Kimberly Page, the CLARO Study Group

**Affiliations:** 1 Department of Internal Medicine, Division of Epidemiology, Biostatistics and Preventive Medicine, University of New Mexico Health Sciences Center, Albuquerque, NM 87131 USA; 2 Department of Medicine, Division of General Internal Medicine, School of Medicine, University of Pittsburgh, 200 Meyran Ave, Suite 300, Pittsburgh, PA 15213 USA; 3 Health Care Division, RAND Corporation, Santa Monica, CA 90417-2038 USA; 4 Grayken Center for Addiction, Boston Medical Center, Boston University, Boston, MA 02118 USA

**Keywords:** Primary care, Opioid use disorder, Mental health, Depression, Post-traumatic stress disorder

## Abstract

**Background:**

Identifying patients in primary care services with opioid use disorder and co-occurring mental health disorders is critical to providing treatment. Objectives of this study were to (1) assess the feasibility of recruiting people to screen in-person for opioid use disorder and co-occurring mental health disorders (depression and/or post-traumatic stress disorder) in primary care clinic waiting rooms in preparation for a randomized controlled trial, and (2) compare results of detecting these disorders by universal in-person screening compared to electronic health record (EHR) diagnoses.

**Methods:**

This cross-sectional feasibility and pilot study recruited participants from four primary care clinics, two rural and two urban, from three health care organizations in New Mexico. Inclusion criteria were adults (≥ 18 years), attending one of the four clinics as a patient, and who spoke English or Spanish. Exclusion criteria were people attending the clinic for a non-primary care visit (e.g., dental, prescription pick up, social support). The main outcomes and measures were (1) recruitment feasibility which was assessed by frequencies and proportions of people approached and consented for in-person screening, and (2) relative differences of detecting opioid use disorder and co-occurring mental health disorders in waiting rooms relative to aggregate EHR data from each clinic, measured by prevalence and prevalence ratios.

**Results:**

Over two-weeks, 1478 potential participants were approached and 1145 were consented and screened (77.5% of patients approached). Probable opioid use disorder and co-occurring mental health disorders were identified in 2.4% of those screened compared to 0.8% in EHR. Similarly, universal screening relative to EHR identified higher proportions of probable opioid use disorder (4.5% vs. 3.4%), depression (17.5% vs. 12.7%) and post-traumatic stress disorder (19.0% vs. 3.6%).

**Conclusions:**

Universal screening for opioid use disorder, depression, and post-traumatic stress disorder was feasible, and identified three times as many patients with these co-occurring disorders compared to EHR. Higher proportions of each condition were also identified, especially post-traumatic stress disorder. Results support that there are likely gaps in identification of these disorders in primary care services and demonstrate the need to better address the persistent public health problem of these co-occurring disorders.

**Supplementary Information:**

The online version contains supplementary material available at 10.1186/s13722-023-00362-5.

## Background

Mental health disorders often co-occur with substance use disorders (SUDs), especially opioid use disorder (OUD), and are often untreated [1, 2]. Non-medical opioid use and co-occurring mental health disorders are linked to an increased risk for OUD and overdose [3–5]. Siloed treatment may result in one or the other disorder going untreated and can have devastating consequences to the individual, their families, and their communities [1, 6]. The primary care (PC) setting has potential for treating these co-occurring disorders as most people visit a primary care provider (PCP) at least once per year [7–9]. A critical first step to engaging patients in PC services for OUD and co-occurring mental health disorders is to identify these patients and characterize their needs. The evidence base for effective pharmacologic and psychological treatment of OUD and these mental health disorders has dramatically increased in recent years [10–13].

Depression, unhealthy drug use, and post-traumatic stress disorder (PTSD) are all common disorders in patients who receive care in PC settings [14]. Screening for these conditions in PC has potential to improve quality of life for patients, contain health care costs, and reduce morbidity that is common when patients have co-occurring conditions [14]. The potential downside however, is that often PC clinics lack resources to implement the necessary structural changes needed (training, education, and operational systems) to ensure appropriate patient follow up. The US Preventive Services Task Force (USPSTF) recommends routine screening of adults for depression [15]. They only recently began recommending screening for unhealthy drug use (including OUD) [16, 17], but there are currently no recommendations for screening for PTSD. Numerous screening instruments have been studied and validated for detecting these disorders; however, a recent USPSTF review estimated that only half of episodes of major depression are identified in PC [18]. The detection of OUD and PTSD, which are screened for less frequently, is likely to be even lower. PTSD is commonly encountered in primary care settings yet is rarely screened for. Although PTSD affects over 10% of adults in the general population [19, 20], it is even more prevalent in primary care settings, affecting up to 32% of patients [21–26]. Primary care patients with PTSD have greater physical complaints including pain, long-term functional impairment, more frequent health care visits, and lower treatment adherence [27].

Co-occurring PTSD and OUD is common but difficult to treat. Poor treatment outcomes may be due to the lack of treatment models that can address both problems simultaneously [28]. In addition, public policies that marginalize substance-using populations increase the likelihood of exposure to traumatic violence and other harmful events. These points highlight the need for research on the effectiveness of public health models that show promise for addressing this complex problem [29]. There is a growing body of literature pointing to high rates of PTSD among those with OUD. This literature consistently indicates a high incidence of PTSD among substance-using populations, with lifetime prevalence rates among SUD individuals ranging from 26 to 52% [30]. Relatively less research has focused on rates of PTSD among individuals with OUD; however, preliminary evidence suggests that rates are equally high. Among OUD populations, 41% have a lifetime history of PTSD and 33% meet criteria for a current PTSD diagnosis, representing the highest rate of PTSD among substance users [31]. We argue that it is important to screen for PTSD because it is equally prevalent as depression in primary care yet is much more likely to go undetected, and it is highly comorbid with OUD as substances are a common way to cope with the consequences of a traumatic event.

We implemented a feasibility and pilot study to assess identification of probable OUD and co-occurring mental health disorders in family practice clinics in New Mexico via universal screening in waiting rooms. The main objectives were to (1) assess the feasibility of implementing universal screening to identify probable OUD, depression, and PTSD among patients in PC clinics to inform recruitment operations for a planned clinical trial, and (2) assess the likelihood of waiting room screening for detecting probable OUD and co-occurring mental health disorders (“observed”) by comparing to electronic health record diagnoses (EHR, “expected”) during the same period. We hypothesized that universal screening in PC for probable OUD and co-occurring mental health disorders would yield a higher number of patients with these disorders relative to what is recorded in the EHR. An exploratory goal was to assess patients’ reports of treatments received for any of these disorders. As pain is often associated with chronic opioid use, OUD, PTSD, and depression [32–34], queries were included to assess self-reported pain in this sample. This study was undertaken in preparation for a multi-site, randomized pragmatic clinical trial that will develop, optimize, and then test a collaborative care intervention strategy intended to improve access, quality, and patient-reported outcomes for individuals in PC with comorbid OUD and depression and/or PTSD [35].

## Methods

### Study design and setting

We administered a cross-sectional survey within four family-practice clinics from three healthcare organizations in New Mexico. Two clinics were located within the Albuquerque city limits and two were in rural areas in Central and Southwestern counties. Three of the clinics were classified as Federally Qualified Health Centers (FQHCs) and the fourth clinic was part of an academic medical center. From October 2018 to September 2019, the clinics saw between 2850 and 5960 unique patients per year. Data for this pilot study were collected in February and March 2020, prior to the SARS-CoV-2 pandemic. We assessed feasibility of recruitment by assessing the number and proportion of people approached for screening and the number and percent of those who consented to screen. The primary objective was to assess identification of probable OUD with probable co-occurring depression and/or PTSD in observed survey data from waiting room screening versus clinical data obtained from the EHR (expected), as well as for each condition individually. ICD-10 codes for diagnoses of OUD, major depressive disorder (MDD), and PTSD were used to identify patients in the EHR during the study period. For these patients, we obtained data related to behavioral health treatment from CPT codes and data on medications prescribed for OUD (Additional File 1: Table S1). All patients identified through the EHR had a diagnosis code noted during the study year and were active patients with the PC clinic during that time. All clinic systems had existing annual screening for depression symptoms using the PHQ-9 that was documented in the electronic medical record; however, clinic systems did not routinely screen for OUD and PTSD prior to the study. Ethical approval for the study was obtained from the RAND Corporation Human Subjects Protection Committee and the University of New Mexico Health Sciences Institutional Review Board. Written informed consent was waived as no patient identifiers were collected and survey responses were anonymous.

### Participants and procedures

Over a two-week study period, research assistants approached people in each of the clinic waiting rooms to screen for eligibility. Adults, ages 18 and older, attending one of the four clinics as a patient, and who spoke English or Spanish were considered eligible and approached. Potential participants were told, “*We are conducting an anonymous survey that will help us test questions for a research project and to better understand the needs of patients at this clinic. If you choose to participate you will receive a $5 merchandise card for your time.*” Those who were eligible and expressed willingness to participate were provided a tablet computer to self-administer a 10-min survey (using REDCap [36]). Participants could skip or decline to answer questions. All who agreed to take the survey were provided with a $5 merchandise card. The survey was available in English and Spanish.

### Measures

Two sources of data were used for this study: aggregate data from each clinic’s EHR and the survey data from waiting room patients. EHR data included: total number of visits, total number of unique patients, and numbers of unique patients with OUD, depression, or PTSD as well as more than one of these diagnoses. We obtained counts from the clinics’ EHRs over a 1-year period (October 1, 2018 – September 30, 2019). Additionally, the clinics provided the aggregate number of unique adult patient PC visits during each clinic’s two-week study period.

In the universal screening survey, probable OUD was screened for using items adapted from the myTAPS screener, a self-administered version of the National Institute on Drug Abuse Tobacco, Alcohol, Prescription medication and other Substance use (TAPS) screener [37]. When used for screening problematic heroin and prescription opioid use, the original TAPS instrument had sensitivity of 0.77 and 0.73 and specificity of 0.99 and 0.98, respectively. Adaptation included (a) restricting to items referring to opioids and some additional questions about prescription pain medications, (b) decomposing Question 1 into component parts to ask about use of prescription pain pills (whether prescribed for participant and if took more than prescribed), and (c) changing the term “prescription pain reliever” to “prescription pain pills” (Additional File 1: Table S2). The Patient Health Questionnaire 8-item version (PHQ-8) was administered to screen for symptoms of depression and probable depression was defined as a summed score of ≥ 10 [38]. The 8-item version, which omits the last question about suicidal ideation and self-injury, was selected due to the pilot nature of the investigation for identifying probable depression via an anonymous, self-administered screener rather than by a provider, thereby limiting intervention by research or clinic staff. The Primary Care PTSD Screen for *DSM-5* (PC-PTSD-5) was used to screen for PTSD and a summed score of ≥ 3 was defined as probable post-traumatic stress disorder [39]. Participants were queried whether in the past 30 days they received: any treatment for substance use disorder or a mental health problem and where they received that treatment. If participants indicated they were taking medication for opioid use disorder, they were also asked to specify which medication, and whether they had been taking the medication for at least the past six months. Participants were asked if they often have pain, and if so, the severity and duration of pain. No adaptations were made to the PHQ-8 or the PC-PTSD-5.

### Statistical analyses

Descriptive statistics from survey responses were calculated for age, gender, language preference, clinic attendance, and pain experience. Aggregate data over the 1-year period from the EHR records was obtained for each clinic and combined. These counts were divided by 26 for an average 2-week estimate for comparability to the waiting room survey data collection period. Medians, 25th and 75th percentiles (Q1 and Q3, respectively), and frequencies and percentages were calculated to summarize data. Prevalence estimates and 95% confidence intervals (CIs) were calculated for the survey responses and EHR samples. Prevalence ratios comparing the survey to EHR estimates and corresponding 95% CIs were calculated.

## Results

### Population identified by universal screening

A total of 1478 people were approached, corresponding to 45.2% of the unique number of patients (3271) that clinics reported seeing during that time period. Of these, 1145 people (77.5%) were eligible (over 18 years of age and a patient at the participating clinic) and consented to participate. The number of eligible and consented participants by clinic were: Clinic A: n = 214 (18.7%); Clinic B: n = 323 (28.2%); Clinic C: n = 352 (30.7%); and Clinic D: n = 256 (22.4%). There were 70 (6.1%) people who started but did not complete the survey. Table 1 presents participant characteristics for those consented and screened. The median (Q1, Q3) age of participants was 50 (33, 62) years, and 65.2% of participants identified as female. The survey was taken in Spanish by 10.7% of participants. The median (Q1, Q3) score for PHQ-8 was 1.0 (0.0, 2.0) and for the PC-PTSD-5 score was 2.0 (1.0, 4.0).

**Table 1:** Characteristics of clinic universal screening participants screened for probable OUD and co-occurring depression and/or PTSD

Characteristic	N (%) or Median (Q1, Q3)
Sample Size	1145
Age in years, Median (Q1, Q3)	50 (33, 62)
Gender, n (%)	
Female	747 (65.2%)
Male	377 (32.9%)
Transgender	10 (0.9%)
Did not identify as Male, Female, or Transgender	3 (0.3%)
Did not respond	8 (0.7%)
Took survey in Spanish, n (%)	122 (10.7%)
Positive for probable opioid use disorder (myTAPS), n (%)	51 (4.5%)
PHQ-8 score (n = 1108^a^)	
Median (Q1, Q3)	1.0 (0.0, 2.0)
Mean (SD)	3.7 (6.2)
Positive for probable depression (PHQ-8 ≥ 10), n (%)	200 (17.5%)
PC-PTSD-5 Score (n = 465^a,b^)	
Median (Q1, Q3)	2.0 (1.0, 4.0)
Mean (SD)	2.4 (1.9)
Positive for probable post-traumatic stress disorder (PC-PTSD-5 ≥ 3), n (%)	218 (19.0%)
Positive for depression & post-traumatic stress disorder, n (%)	102 (8.9%)
Positive for probable opioid use disorder + depression and/or post-traumatic stress disorder, n (%)	27 (2.4%)
Positive for probable opioid use disorder + depression + post-traumatic stress disorder, n (%)	10 (0.9%)
Often have pain, n (%)	598 (52.2%)
Pain severity, n (%)	
Mild	107 (17.9%)
Moderate	336 (56.2%)
Severe	151 (25.3%)
Did not respond	4 (0.7%)
Length of time with pain, n (%)	
< 1 week	32 (5.4%)
1 week—2 months	52 (8.7%)
2–6 months	52 (8.7%)
> 6 months	415 (69.4%)
Don’t know	41 (6.9%)
Did not respond	6 (1.0%)

*myTAPS* self-administered Tobacco, Alcohol, Prescription medication and other Substance use screener, *PHQ-8* Patient Health Questionnaire 8-item version, *PC-PTSD-5* Primary Care PTSD Screen for *DSM-5*

*Q1, Q3* 1st and 3rd quartiles

^a^37 of 1145 (3.2%) participants did not answer any PHQ-8 or PC-PTSD-5 questions

^b^465 of 1145 (40.6%) participants responded “yes” to the question, “Sometimes things happen to people that are unusually or especially frightening, horrible, or traumatic [examples given]. Have you ever experienced this kind of event?” Only these participants were asked five additional questions to yield a PC-PTSD-5 summed score

Overall, 4.5% (51/1145) of participants surveyed had probable OUD, 17.5% (200/1145) screened positive for probable depression, and 19% (218/1145) for probable PTSD. There were 110 (9.6%) participants who had both probable depression and PTSD. Probable OUD and co-occurring mental health disorders were identified in 27 (2.4%) participants with 19 (70.4%) who were female and 3 (11.1%) who took the survey in Spanish. About half (52.2%) of all participants reported having frequent pain; 25.3% of them reported severe pain and 56.2% reported moderate pain, most of the time. Pain was reported as ongoing for more than 6 months in 69.4% of participants who reported frequent pain. When asked to reflect on the past 3 months use of prescription pain pills, of all participants, 55 (4.8%) reported using pain pills not prescribed for them, and 20 (1.7%) reported taking more pills than prescribed. There were 22 (1.9%) participants who reported using heroin in the past 3 months.

Among the group with probable OUD and co-occurring mental health disorders (n = 27), recent (past three month) prescription pain pill use was reported by 18 (66.7%), and recent heroin use by 12 (44.4%). Thirteen (48.1%) reported not getting any treatment for these conditions in the past 30 days; six (22.2%) reported counseling/therapy only, three (11.1%) reported medication only, and five (18.5%) reported receiving both medication and counseling/therapy. Among those receiving counseling/therapy, 54.5% (6 of 11 participants) were receiving it at the same clinic where they interviewed. Most (6 of 8 participants, 75.0%) of those who reported receiving medication for opioid use or mental health disorders had it prescribed from the same clinic.

### Comparison of universal waiting room screening to EHR data

In comparison with the number of patients with diagnosed OUD, depression, and/or post-traumatic stress disorder who were identified in clinic EHRs over a 2-week period (based on 2018–2019 clinic flow estimates), the universal screening survey identified a slightly higher proportion with probable OUD than expected (Table 2). Compared to the EHR, universal screening identified approximately 1.4 times the number of patients with probable depression and 5.2 times the number of patients with probable PTSD. There were nearly three times as many patients identified with probable OUD and co-occurring mental health disorders in the waiting room sample compared to EHR. EHR indicators of patients’ primary language identified that 18.5% were monolingual Spanish speakers; however, 10.7% of our waiting room sample selected to take the survey in Spanish.

**Table 2:** Prevalence of probable OUD, mental health disorders, and language in universal screening participants versus EHR

	Universal Screening Survey (N = 1478)^a^		Clinic EHR Estimates (N = 740)^b^		Prevalence Ratio (95% CI)^e^
	n (%)	95% CI	n (%)	95% CI	
Total participants consented	1145 (77.5%)^c^	75.3%, 79.6%	–	–	–
Total participants with probable opioid use disorder	51 (4.5%)^d^	3.3%, 5.6%	25 (3.4%)	2.1%, 4.7%	1.32 (0.8, 2.11)
Total participants with probable depression	200 (17.5%)^d^	15.3%, 19.7%	94 (12.7%)	10.3%, 15.1%	**1.38 (1.10, 1.73)**
Total participants with probable post-traumatic stress disorder	218 (19.0%)^d^	16.8%, 21.3%	27 (3.6%)	2.3%, 5.0%	**5.22 (3.54, 7.70)**
Total participants with probable opioid use disorder + co-occurring mental health disorders (depression and/or post-traumatic stress disorder)	27 (2.4%)^d^	1.5%, 3.2%	6 (0.8%)	0.2%, 1.5%	**2.91 (1.21, 7.01)**
Total number monolingual Spanish/took survey in Spanish	122 (10.7%)^d^	8.9%, 12.4%	137 (18.5%)	15.7%, 21.3%	**0.58 (0.46, 0.72)**

*EHR* electronic health record

^a^For the Universal screening study, this may be an undercount as they were all patients the research staff correctly approached for potential participation. Correctly means the person was age 18 + and was at the clinic for a primary care visit. Some patients may have been missed

^b^Data collected from the EHR was collected over a 1-year period from October 1, 2018 to September 30, 2019. Counts have been divided by 26 to estimate an average 2-week period for comparison to the pilot data

^c^Denominator is patients correctly approached, N = 1478

^d^Denominator is patients consented, N = 1145

^e^Bolded values indicate prevalence ratio is statistically significant, assuming a type I error level of 0.05

## Discussion

Overall, in-person screening identified a nearly three-fold higher proportion of patients (2.4%) with probable OUD and co-occurring mental health disorders compared to the EHR (0.8%). We also identified a higher proportion of each condition separately (OUD, depression, and PTSD) with the survey. To our knowledge the prevalence of OUD and co-occurring mental health disorders has not been quantified and compared using a universal screening approach in primary care clinics in other studies. It is not surprising that EHR data would underestimate these conditions as patients may not disclose symptoms or problems to providers in association with discomfort, poor help-seeking intention, trust, shame, or stigma [40–43]. Underdiagnosis of major depressive disorder is more common in racial and ethnic minority populations in the US, and the population of New Mexico has a high proportion of Hispanic patients [44]. The most striking difference between observed and expected prevalence was for probable PTSD, which was over five-fold higher in the clinic sample than the EHR sample. All of the participating clinics routinely screen for depression, and OUD has become a more visible issue regionally and nationally, but patients are not routinely screened for PTSD. Furthermore, avoidance of trauma reminders is a common PTSD-related symptom [45], making it understandable that patients with PTSD may avoid discussing their symptoms and experiences with providers [25]. The anonymity of the survey likely reduced non-disclosure that can occur in clinical practice regarding sensitive issues like mental health status [46, 47]. Under-reporting of substance use in PC is well recognized and has been shown to vary by substance used, but is often found with opioids [47, 48]. It remains important to implement screening methods for these conditions that minimize judgment and stigma.

Our study also found large variability in treatment for OUD and co-occurring mental health disorders relative to probable diagnoses. In the universal screening sample, among those with probable OUD and co-occurring mental health disorders, almost half (46.2%) reported not receiving any treatment, and only one in five participants was receiving both medication and counseling for at least one of the conditions. Other studies have also shown low rates of treatment for OUD in PC clinics [49, 50] despite strong evidence that methadone and buprenorphine-naloxone treatment is more effective than abstinence-based treatment [10, 51]. PCPs have pointed to a lack of integrated behavioral health providers as a common reason for not prescribing MOUDs [52]. Hallgren et al. [50] provided support for this in their study of EHR data from patients attending 21 PC clinics: those with OUD diagnoses were more likely to receive medications if they were seen in clinics with co-located non-physician behavioral health specialists.

Our results also show missed opportunities for universal screening of probable OUD and co-occurring mental health disorders in PC. Currently, the USPSTF recommends routine screening of adults for depression [15] and for SUD, including OUD [16], but not for PTSD possibly due to a lack of knowledge surrounding the prevalence of the condition and the difficulties associated with treating it. Each of these conditions separately was also more common in the screened sample. Lastly, considering the high prevalence of chronic pain reported in our waiting room screening sample (52%), of whom 81% reported moderate or severe pain, and the potential for exacerbated negative physical and psychological outcomes in people with intersecting pain and mental health problems, more options are needed to identify and treat these patients.[33, 34]. The difficulties encountered by busy PC clinics in identifying patients with co-occurring substance use and mental disorders, and then linking those patients to appropriate care, are significant and understandable. It is critical to ensure that adequate services, including evidence-based treatment, are available to patients in whom these problems are identified, either in the PC setting or by referral. For example, this study was conducted as part of planning for a randomized controlled trial of collaborative care for OUD and co-occurring mental health disorders [35]. Collaborative care is a team-based PC model for managing behavioral health in which a care manager helps ensure patients are identified and linked to evidence-based and measurement-based care with PCPs and behavioral health clinicians, all overseen by a psychiatric consultant. Collaborative care has demonstrated improved care access and outcomes for major depressive disorder, PTSD, and OUD, each separately [21, 53–56], as well as for alcohol use disorder [54]. Our trial will test whether it produces the same results for patients with OUD and co-occurring mental health disorders [35]. As hypothesized, the pilot study demonstrated the feasibility of identifying our target patient population through universal screening.

This study has several potential limitations including the cross-sectional design and self-reported data obtained from universal screening. Recall and reporting bias can occur when assessing sensitive conditions, resulting in underestimation of the prevalence estimates. However, since the survey was anonymous this limitation may have been minimized. The consistency of our results showing significant differences in observed versus expected prevalence of disorders also suggests that reporting bias was low. Our sample is not likely to be representative of PC patient populations in other locations; it was limited to English and Spanish speaking patients at PC clinics in New Mexico, three of which were FQHCs. Strengths of the results include the relatively large number of patients accessed over the 2-week survey period and the use of validated screening instruments. While our research assistants reached approximately 45% of adults visiting the primary care clinics, we demonstrated acceptability of the screening questions via the high completion rate (93.9%). Assessing these conditions without anonymizing questions and providing results to the primary care provider has potential to be effective. The participating clinics in this study have a strong community presence and have a known positive, non-judgmental approach to substance use, substance use treatment, and mental health; qualities that could enhance self-reporting. One approach could be with self-administered screeners given to every patient at check-in on a regular basis. For example, the PHQ-9 is already administered to every patient at these study clinics on an annual basis.

## Conclusions

This study helps quantify the potential extent of diagnostic and treatment service gaps for OUD and co-occurring mental health disorders in PC settings serving rural and socioeconomically disadvantaged patients in New Mexico. Rates of these disorders in these settings are generally higher than what is documented in the EHR (as seen in this study and in previous studies in PC and integrated health systems) [49, 50]. Undertreatment of OUD and mental health disorders remain a persistent public health problem [57, 58]. And, as the COVID-19 pandemic has been associated with increases in psychological distress, adverse mental health conditions, and opioid-related overdose, the imperative to address these conditions is higher than ever [59–62]. This study demonstrates that meaningful identification of these disorders is feasible via a universal screening approach and may help identify patients who may otherwise go undiagnosed. It also highlights the need for additional research to fully characterize the prevalence of OUD and co-occurring mental health disorders and their treatments among PC patients, and examine methods to diagnose, engage, and provide effective treatments. Ultimately, to improve outcomes, screening in PC settings needs to be linked with education and training for clinical staff, as well as operational processes that ensure effective follow-up.

## Supplementary Information

**Additional file 1: Table S1.** Electronic health record patient identification criteria for comparator cohort, October 2018 – September 2019. **Table S2.** Questions asked on the universal screening survey for probable OUD, depression, PTSD, treatment, and pain.

## Data Availability

The datasets generated and analyzed during this study are not publicly available due to the sensitive nature of the data. They can be made available from the corresponding author on reasonable request and with execution of appropriate Data Use Agreements.

## Abbreviations

CIs: Confidence intervals
CPT: Current Procedural Terminology
DSM-5: Diagnostic and Statistical Manual of Mental Health Disorders, 5^th^ Edition
EHR: Electronic health records
FQHC: Federally qualified health center
ICD-10: International Classification of Diseases, Tenth Revision
MDD: Major depressive disorder
MOUD: Medication for opioid use disorder
OUD: Opioid use disorder
PC: Primary care
PCP: Primary care provider
PC-PTSD-5: Primary Care PTSD Screen for DSM-5
PHQ-8: Patient Health Questionnaire-8 item version
PTSD: Post-traumatic stress disorder
Q1: Quartile 1, or 25th percentile
Q3: Quartile 3, or 75th percentile
SUD: Substance use disorder
TAPS: National Institute on Drug Abuse Tobacco, Alcohol, Prescription medication and other Substance use screener
USPSTF: United States Preventive Services Task Force

## References

[1] Han B, Compton WM, Blanco C, Colpe LJ (2017). Prevalence, treatment, and unmet treatment needs Of US Adults with mental health and substance use disorders. Health Aff.

[2] Compton WM, Thomas YF, Stinson FS, Grant BF (2007). Prevalence, correlates, disability, and comorbidity of DSM-IV drug abuse and dependence in the United States: results from the national epidemiologic survey on alcohol and related conditions. Arch Gen Psychiatry.

[3] Drug Overdose Deaths. 2021. https://www.cdc.gov/drugoverdose/data/statedeaths.html. Accessed 13 Apr 2021.

[4] Compton WM, Jones CM, Baldwin GT (2016). Relationship between nonmedical prescription-opioid use and Heroin use. N Engl J Med.

[5] Jones CM (2013). Heroin use and heroin use risk behaviors among nonmedical users of prescription opioid pain relievers - United States, 2002–2004 and 2008–2010. Drug Alcohol Depend.

[6] Drug Overdose Deaths. 2021. https://www.cdc.gov/drugoverdose/data/statedeaths.html. Accessed 8 Apr 2021.

[7] Crowley RA, Kirschner N (2015). Health and Public Policy Committee of the American College of Physicians. The integration of care for mental health, substance abuse, and other behavioral health conditions into primary care: executive summary of an American College of Physicians position paper. Ann Intern Med.

[8] Steinberg J, Azofeifa A, Sigounas G (2019). Mobilizing primary care to address the opioid use disorder treatment gap. Public Health Rep.

[9] Hooker SA, Sherman MD, Lonergan-Cullum M, Sattler A, Liese BS, Justesen K (2020). Mental health and psychosocial needs of patients being treated for opioid use disorder in a primary care residency clinic. J Prim Care Community Health.

[10] National Academies of Sciences, Engineering, and Medicine. Medications for opioid use disorder save lives. Washington, DC: The National Academies Press. 2019. 10.17226/25310. 30896911

[11] Pampallona S, Bollini P, Tibaldi G, Kupelnick B, Munizza C (2004). Combined pharmacotherapy and psychological treatment for depression: a systematic review. Arch Gen Psychiatry.

[12] Puetz TW, Youngstedt SD, Herring MP (2015). Effects of pharmacotherapy on combat-related PTSD, anxiety, and depression: a systematic review and meta-regression analysis. PLoS ONE.

[13] Farah WH, Alsawas M, Mainou M, Alahdab F, Farah MH, Ahmed AT (2016). Non-pharmacological treatment of depression: a systematic review and evidence map. Evid Based Med.

[14] Mulvaney-Day N, Marshall T, Downey Piscopo K, Korsen N, Lynch S, Karnell LH (2018). Screening for behavioral health conditions in primary care settings: a systematic review of the literature. J Gen Intern Med.

[15] Siu AL, Bibbins-Domingo K, Grossman DC, Baumann LC, Davidson KW, US Preventive Services Task Force (USPSTF) (2016). Screening for depression in adults: US preventive services task force recommendation statement. JAMA.

[16] Saitz R (2020). Screening for unhealthy drug use: neither an unreasonable idea nor an evidence-based practice. JAMA: J Am Med Assoc.

[17] Draft research plan: Depression, anxiety, and suicide risk in adults, including pregnant and postpartum persons: Screening. 2020. https://www.uspreventiveservicestaskforce.org/uspstf/document/draft-research-plan/screening-depression-anxiety-suicide-risk-adults. Accessed 24 Jun 2022.

[18] O’Connor EA, Whitlock EP, Beil TL, Gaynes BN (2009). Screening for depression in adult patients in primary care settings: a systematic evidence review. Ann Intern Med.

[19] Schnurr PP, Spiro A, Paris AH (2000). Physician-diagnosed medical disorders in relation to PTSD symptoms in older male military veterans. Health Psychol.

[20] Kessler RC, Chiu WT, Demler O, Merikangas KR, Walters EE (2005). Prevalence, severity, and comorbidity of 12-month DSM-IV disorders in the National Comorbidity Survey Replication. Arch Gen Psychiatry.

[21] Meredith LS, Eisenman DP, Han B, Green BL, Kaltman S, Wong EC (2016). Impact of collaborative care for underserved patients with PTSD in primary care: a randomized controlled trial. J Gen Intern Med.

[22] Stein MB, McQuaid JR, Pedrelli P, Lenox R, McCahill ME (2000). Posttraumatic stress disorder in the primary care medical setting. Gen Hosp Psychiatry.

[23] Gillock KL, Zayfert C, Hegel MT, Ferguson RJ (2005). Posttraumatic stress disorder in primary care: prevalence and relationships with physical symptoms and medical utilization. Gen Hosp Psychiatry.

[24] Magruder KM, Frueh BC, Knapp RG, Davis L, Hamner MB, Martin RH (2005). Prevalence of posttraumatic stress disorder in Veterans Affairs primary care clinics. Gen Hosp Psychiatry.

[25] Liebschutz J, Saitz R, Brower V, Keane TM, Lloyd-Travaglini C, Averbuch T (2007). PTSD in urban primary care: high prevalence and low physician recognition. J Gen Intern Med.

[26] Spottswood M, Davydow DS, Huang H (2017). The prevalence of posttraumatic stress disorder in primary care: a systematic review. Harv Rev Psychiatry.

[27] Possemato K (2011). The current state of intervention research for posttraumatic stress disorder within the primary care setting. J Clin Psychol Med Settings.

[28] Fareed A, Eilender P, Haber M, Bremner J, Whitfield N, Drexler K (2013). Comorbid posttraumatic stress disorder and opiate addiction: a literature review. J Addict Dis.

[29] Dahlby L, Kerr T (2020). PTSD and opioid use: implications for intervention and policy. Subst Abuse Treat Prev Policy.

[30] Roberts NP, Roberts PA, Jones N, Bisson JI (2015). Psychological interventions for post-traumatic stress disorder and comorbid substance use disorder: a systematic review and meta-analysis. Clin Psychol Rev.

[31] Ecker AH, Hundt N (2018). Posttraumatic stress disorder in opioid agonist therapy: a review. Psychol Trauma.

[32] Tsui JI, Lira MC, Cheng DM, Winter MR, Alford DP, Liebschutz JM (2016). Chronic pain, craving, and illicit opioid use among patients receiving opioid agonist therapy. Drug Alcohol Depend.

[33] IsHak WW, Wen RY, Naghdechi L, Vanle B, Dang J, Knosp M (2018). Pain and depression: a systematic review. Harv Rev Psychiatry.

[34] Beck JG, Clapp JD (2011). A different kind of co-morbidity: Understanding posttraumatic stress disorder and chronic pain. Psychol Trauma.

[35] Meredith LS, Komaromy MS, Cefalu M, Murray-Krezan C, Page K, Osilla KC (2021). Design of CLARO (collaboration leading to addiction treatment and recovery from other stresses): a randomized trial of collaborative care for opioid use disorder and co-occurring depression and/or posttraumatic stress disorder. Contemp Clin Trials.

[36] Harris PA, Taylor R, Thielke R, Payne J, Gonzalez N, Conde JG (2009). Research electronic data capture (REDCap)–a metadata-driven methodology and workflow process for providing translational research informatics support. J Biomed Inform.

[37] Adam A, Schwartz RP, Wu L-T, Subramaniam G, Laska E, Sharma G (2019). Electronic self-administered screening for substance use in adult primary care patients: feasibility and acceptability of the tobacco, alcohol, prescription medication, and other substance use (myTAPS) screening tool. Addict Sci Clin Pract.

[38] Kroenke K, Strine TW, Spitzer RL, Williams JBW, Berry JT, Mokdad AH (2009). The PHQ-8 as a measure of current depression in the general population. J Affect Disord.

[39] Prins A, Bovin MJ, Smolenski DJ, Marx BP, Kimerling R, Jenkins-Guarnieri MA (2016). The primary care PTSD screen for DSM-5 (PC-PTSD-5): development and evaluation within a veteran primary care sample. J Gen Intern Med.

[40] Walters K, Buszewicz M, Weich S, King M (2008). Help-seeking preferences for psychological distress in primary care: effect of current mental state. Br J Gen Pract.

[41] Kravitz RL, Paterniti DA, Epstein RM, Rochlen AB, Bell RA, Cipri C (2011). Relational barriers to depression help-seeking in primary care. Patient Educ Couns.

[42] Clement S, Schauman O, Graham T, Maggioni F, Evans-Lacko S, Bezborodovs N (2015). What is the impact of mental health-related stigma on help-seeking? A systematic review of quantitative and qualitative studies. Psychol Med.

[43] Yakeley J (2018). Shame, culture and mental health. Nord J Psychiatry.

[44] Shao Z, Richie WD, Bailey RK (2016). Racial and ethnic disparity in major depressive disorder. J Racial Ethn Health Disparities.

[45] McMillen JC, North CS, Smith EM (2000). What parts of PTSD are normal: intrusion, avoidance, or arousal? Data from the Northridge, California, earthquake. J Trauma Stress.

[46] Bell RA, Franks P, Duberstein PR, Epstein RM, Feldman MD, Fernandez Y, Garcia E (2011). Suffering in silence: reasons for not disclosing depression in primary care. Ann Fam Med..

[47] McNeely J, Adam A, Rotrosen J, Wakeman SE, Wilens TE, Kannry J (2021). Comparison of methods for alcohol and drug screening in primary care clinics. JAMA Netw Open.

[48] Bone C, Gelberg L, Vahidi M, Leake B, Yacenda-Murphy J, Andersen RM (2016). Under-reporting of risky drug use among primary care patients in federally qualified health centers. J Addict Med.

[49] Lapham G, Boudreau DM, Johnson EA, Bobb JF, Matthews AG, McCormack J (2020). Prevalence and treatment of opioid use disorders among primary care patients in six health systems. Drug Alcohol Depend.

[50] Hallgren KA, Witwer E, West I, Baldwin L-M, Donovan D, Stuvek B (2020). Prevalence of documented alcohol and opioid use disorder diagnoses and treatments in a regional primary care practice-based research network. J Subst Abuse Treat.

[51] Mattick RP, Breen C, Kimber J, Davoli M (2014). Buprenorphine maintenance versus placebo or methadone maintenance for opioid dependence. Cochrane Database Syst Rev.

[52] Hutchinson E, Catlin M, Andrilla CHA, Baldwin L-M, Rosenblatt RA (2014). Barriers to primary care physicians prescribing buprenorphine. Ann Fam Med.

[53] Coventry PA, Hudson JL, Kontopantelis E, Archer J, Richards DA, Gilbody S (2014). Characteristics of effective collaborative care for treatment of depression: a systematic review and meta-regression of 74 randomised controlled trials. PLoS ONE.

[54] Watkins KE, Ober AJ, Lamp K, Lind M, Setodji C, Osilla KC (2017). Collaborative care for opioid and alcohol use disorders in primary care: the SUMMIT randomized clinical trial. JAMA Intern Med.

[55] Woltmann E, Grogan-Kaylor A, Perron B, Georges H, Kilbourne AM, Bauer MS (2012). Comparative effectiveness of collaborative chronic care models for mental health conditions across primary, specialty, and behavioral health care settings: systematic review and meta-analysis. Am J Psychiatry.

[56] Brackett CD, Duncan M, Wagner JF, Fineberg L, Kraft S (2021). Multidisciplinary treatment of opioid use disorder in primary care using the collaborative care model. Subst Abus.

[57] Blanco C, Volkow ND (2019). Management of opioid use disorder in the USA: present status and future directions. Lancet.

[58] Kessler RC, Demler O, Frank RG, Olfson M, Pincus HA, Walters EE (2005). Prevalence and treatment of mental disorders, 1990 to 2003. N Engl J Med.

[59] Holland KM, Jones C, Vivolo-Kantor AM, Idaikkadar N, Zwald M, Hoots B (2021). Trends in US emergency department visits for mental health, overdose, and violence outcomes before and during the COVID-19 pandemic. JAMA Psychiat.

[60] Czeisler MÉ, Lane RI, Petrosky E, Wiley JF, Christensen A, Njai R (2020). Mental health, substance use, and suicidal ideation during the COVID-19 pandemic - United States, June 24–30, 2020. MMWR Morb Mortal Wkly Rep.

[61] Addiction Policy Forum. COVID-19 pandemic impact on patients, families & individuals in recovery from a SUD. APF. 2020. https://www.addictionpolicy.org/post/covid-19-pandemic-impact-on-patients-families-individuals-in-recovery-fromsubstance-use-disorder. Accessed 26 Jan 2022.

[62] Soares WE, Melnick ER, Nath B, D’Onofrio G, Paek H, Skains RM (2022). Emergency department visits for nonfatal opioid overdose during the COVID-19 pandemic across six US health care systems. Ann Emerg Med.

