# Supplementary material for: Screening for opioid use disorder and co-occurring depression and post-traumatic stress disorder in primary care in New Mexico
Source: Addict Sci Clin Pract. 2023 Jan 27;18:6. doi: 10.1186/s13722-023-00362-5 (PMC9881516; doi:10.1186/s13722-023-00362-5)
Supplement: Supplementary file 1 — Additional file 1: Table S1. Electronic health record patient identification criteria for comparator cohort, October 2018 – September 2019. Table S2. Questions asked on the universal screening survey for probable OUD, depression, PTSD, treatment, and pain. [file 13722_2023_362_MOESM1_ESM.docx]

**Additional file 1: Table S1.** Electronic health record patient identification criteria for comparator cohort, October 2018 – September 2019.

| **Opioid Use Disorder (OUD) ^1^** | | **Major Depressive Disorder (MDD) ^1^** | | **Post-Traumatic Stress Disorder (PTSD) ^1^** | | **Behavioral Health Treatment ^2^** | | **Medications for OUD ^2^** |
| --- | --- | --- | --- | --- | --- | --- | --- | --- |
| *ICD-10* | *Description* | *ICD-10* | *Description* | *ICD-10* | *Description* | *CPT Code* | *Description* |  |
| F11.10 | Opioid abuse, uncomplicated | F32.0 | Major depressive disorder, single episode, mild | F43.10 | Post-traumatic stress disorder, unspecified | 90832 | Psychotherapy, 30 minutes with patient | buprenorphine |
| F11.120 | Opioid abuse with intoxication, uncomplicated | F32.1 | Major depressive disorder, single episode, moderate | F43.11 | Post-traumatic stress disorder, acute | 90833 | Psychotherapy, 30 minutes with patient when performed with an evaluation and management service | Suboxone |
| F11.121 | Opioid abuse with intoxication delirium | F32.2 | Major depressive disorder, single episode, severe without psychotic features | F43.12 | Post-traumatic stress disorder, chronic | 90834 | Psychotherapy, 45 minutes with patient | Subutex |
| F11.122 | Opioid abuse with intoxication with perceptual disturbance | F32.4 | Major depressive disorder, single episode, in partial remission |  |  | 90836 | Psychotherapy, 45 minutes with patient when performed with an evaluation and management service | Sublocade |
| F11.129 | Opioid abuse with intoxication, unspecified | F32.9 | Major depressive disorder, single episode, unspecified |  |  | 90837 | Psychotherapy, 60 minutes with patient | Zubsolv |
| F11.14 | Opioid abuse with opioid-induced mood disorder | F33.0 | Major depressive disorder, recurrent, mild |  |  | 90838 | Psychotherapy, 60 minutes with patient when performed with an evaluation and management service | buprenorphine-naloxone (could be written differently depending on the EHR, e.g., buprenorphine/naloxone) |
| F11.150 | Opioid abuse with opioid-induced psychotic disorder with delusions | F33.1 | Major depressive disorder, recurrent, moderate |  |  | 90839 | Psychotherapy for crisis; first 60 minutes |  |
| F11.151 | Opioid abuse with opioid-induced psychotic disorder with hallucinations | F33.2 | Major depressive disorder, recurrent severe without psychotic features |  |  | 90853 | Group psychotherapy |  |
| F11.159 | Opioid abuse with opioid-induced psychotic disorder, unspecified | F33.41 | Major depressive disorder, recurrent, in partial remission |  |  |  |  |  |
| F11.181 | Opioid abuse with opioid-induced sexual dysfunction | F33.9 | Major depressive disorder, recurrent, unspecified |  |  |  |  |  |
| F11.182 | Opioid abuse with opioid-induced sleep disorder | F34.1 | Dysthymic disorder |  |  |  |  |  |
| F11.188 | Opioid abuse with other opioid-induced disorder |  |  |  |  |  |  |  |
| F11.19 | Opioid abuse with unspecified opioid-induced disorder |  |  |  |  |  |  |  |
| F11.20 | Opioid dependence, uncomplicated |  |  |  |  |  |  |  |
| F11.220 | Opioid dependence with intoxication, uncomplicated |  |  |  |  |  |  |  |
| F11.221 | Opioid dependence with intoxication delirium |  |  |  |  |  |  |  |
| F11.222 | Opioid dependence with intoxication with perceptual disturbance |  |  |  |  |  |  |  |
| F11.229 | Opioid dependence with intoxication, unspecified |  |  |  |  |  |  |  |
| F11.23 | Opioid dependence with withdrawal |  |  |  |  |  |  |  |
| F11.24 | Opioid dependence with opioid-induced mood disorder |  |  |  |  |  |  |  |
| F11.250 | Opioid dependence with opioid-induced psychotic disorder with delusions |  |  |  |  |  |  |  |
| F11.251 | Opioid dependence with opioid-induced psychotic disorder with hallucinations |  |  |  |  |  |  |  |
| F11.259 | Opioid dependence with opioid-induced psychotic disorder, unspecified |  |  |  |  |  |  |  |
| F11.281 | Opioid dependence with opioid-induced sexual dysfunction |  |  |  |  |  |  |  |
| F11.282 | Opioid dependence with opioid-induced sleep disorder |  |  |  |  |  |  |  |
| F11.288 | Opioid dependence with other opioid-induced disorder |  |  |  |  |  |  |  |
| F11.29 | Opioid dependence with unspecified opioid-induced disorder |  |  |  |  |  |  |  |
| F11.93 | Opioid use, unspecified with withdrawal |  |  |  |  |  |  |  |
| ^1^ Patients were counted if they had one or more of these qualifying diagnoses.  ^2^ Behavioral health treatment information and prescribed medications were obtained only for those with a diagnosis of OUD, MDD, or PTSD. | | | | | | | | |

**Additional file 1: Table S2.** Questions asked on the universal screening survey for probable OUD, depression, PTSD, treatment, and pain.

| Construct | Question or Instrument | Scoring |
| --- | --- | --- |
| Opioid use disorder, past 30 days | Adapted from myTAPS: |  |
|  | (Q1) Had they taken any prescription pain pills, even just once | Responses:   - “Yes” - “No”   Probable opioid use disorder = “Yes” to at least one of Q2, Q3, Q5, or Q6 |
|  | (Q1a) If Q1=”Yes”, were any prescription pain pills taken not prescribed for them |  |
|  | (Q1b) If Q1=”Yes”, Did they take more prescription pain pills than prescribed |  |
|  | (Q2) Had they had tried and failed to control their use of prescription pain pills |  |
|  | (Q3) Had anyone told them they were worried about their use of prescription pain pills |  |
|  | (Q4) Had they used heroin |  |
|  | (Q5) Had they had tried and failed to control their heroin use |  |
|  | (Q6) Had anyone told them they were worried about their use of heroin |  |
|  | | |
| Depression symptoms, last two weeks | PHQ-8 (8 questions) | Responses:   - “Not at all”=0 - “Several days”=1 - “More than half the days”=2 - “Nearly every day”=3   Probable depression = summed score ≥ 10 |
|  | | |
| Post-traumatic stress disorder symptoms, last one month | PC-PTSD-5 (If participant responds yes to first question, then remaining 5 questions are asked and a score is calculated from those). | Responses:   - ”Yes”=1 - ”No”=0   Probable post-traumatic stress disorder = Summed score ≥ 3 |
|  |  |  |
| Treatment for opioid use disorder, depression, and/or post-traumatic stress disorder, past 30 days | (Q1) Received any treatment for substance use disorder or a mental health problem | Responses:   - “Medication” - “Therapy/ Counseling” - “Both” - “Neither” |
|  | (Q2) Where they received that treatment | Responses:   - “This clinic” - “A different clinic in the same health system” - “A different health system” - “Without a prescription” (for medication only) |
|  | (Q3) If taking medication for opioid use disorder, asked if they were taking… | Responses:   - “Buprenorphine or buprenorphine+naloxone” - “Injectable naltrexone” - “Methadone” - “Other/Don’t know” |
|  | (Q4) If taking buprenorphine or buprenorphine+naloxone, injectable naltrexone, or methadone, had they been taking it for at least the last six months | Responses:   - “Yes” - “No” |
|  | | |
| Pain | (Q1) Whether they often have pain | Responses:   - “Yes” - “No” |
|  | (Q1a) If Q1=”Yes”, severity of pain | Responses:   - “Mild” - “Moderate” - “Severe” |
|  | (Q1b) If Q1=”Yes”, how long have they had pain | Responses:   - “Less than one week” - “Between one week and two months” - “Between two and six months” - “More than six months” - “Don’t know” |
